# Developing a core outcome set for paediatric wrist fractures: a systematic review of prior outcomes

**DOI:** 10.1302/2633-1462.15.BJO-2020-0007.R1

**Published:** 2020-09-01

**Authors:** Benjamin Thomas Crosby, Abolfazl Behbahani, Olivia Olujohungbe, Ben Cottam, Daniel Perry

**Affiliations:** 1 University of Liverpool, Liverpool, UK; 2 University of Plymouth, Plymouth, England

**Keywords:** Outcome, Wrist fracture, Paediatric

## Abstract

**Objectives:**

This review aims to summarize the outcomes used to describe effectiveness of treatments for paediatric wrist fractures within existing literature.

**Method:**

We searched the Cochrane Library, Scopus, and Ovid Medline for studies pertaining to paediatric wrist fractures. Three authors independently identified and reviewed eligible studies. This resulted in a list of outcome domains and outcomes measures used within clinical research. Outcomes were mapped onto domains defined by the COMET collaborative.

**Results:**

Our search terms identified 4,262 different papers. Screening of titles excluded 2,975, leaving 1,287 papers to be assessed for eligibility. Of this 1,287, 30 studies were included for full analysis. Overall, five outcome domains, 16 outcome measures, and 28 measurement instruments were identified as outcomes within these studies. 24 studies used at least one measurement pertaining to the physiological/clinical outcome domain. The technical, life impact, and adverse effect domains were recorded in 23, 20, and 11 of the studies respectively. Within each domain it was common for different measurement instruments to be used to assess each outcome measure. The most commonly reported outcome measures were range of movement, a broad array of “radiological measures” and pain intensity, which were used in 24, 23, and 12 of the 30 studies.

**Conclusion:**

This study highlights the heterogeneity in outcomes reported within clinical effectiveness studies of paediatric wrist fractures. We provided an overview of the types of outcomes reported in paediatric wrist fracture studies and identified a list of potentially relevant outcomes required for the development of a core outcome set.

## Introduction

Wrist fractures are extremely common within a paediatric population. One-third of children suffer at least one fracture before the age of 17 years,^1^ and fractures represent 9% of all childhood injuries that present to health services.^2^ Of these injuries, distal radii fractures are the most frequent.

Despite the frequency of these injuries, optimal treatment strategies remain controversial. The treatment of fractures follows the basic principles of adequately restoring the anatomy, holding the bone to facilitate healing and encouraging rehabilitation. However, due to the remodelling capacity of children's bone is such that perfect anatomical correction of a fracture is not often not required, as the bone will return to a normal shape as it grows. The optimal treatment of fractures and the decision to operate is informed by research; however, the interpretation of such evidence is often hampered by the heterogeneity of study design and the lack of an agreed set of outcomes. This variation often acts as a barrier when drawing comparisons between studies. Due to these problems there is currently significant variation within the practice habits and management of paediatric wrist fractures.^3^ Homogenising care is consistent with the UK-wide National Health Service (NHS) agenda to eradicate unnecessary variation, through the vehicle of ‘Getting It Right First Time (GIRFT)’.^4^

One way to reduce this variation is through the development of a core outcome set (COS), which represent a minimum required data set for randomized controlled trials of certain conditions ^5,6^ and aim to reduce heterogeneity in both research and clinical practice. They allow for easy comparison between studies, facilitate meta-analyses, improve the accuracy of data interpretation and reduce outcome reporting bias.^7^

The Core Outcome Measures in Effective Trials (COMET) initiative was set up in January 2010 and aims to facilitate the development and application of COSs in order to overcome outcome reporting variation.^8^

This systematic review forms the foundations for the development of a COS for the management of paediatric wrist fractures. This review aims to collate and summarize the outcome domains utilized to report the outcomes of paediatric wrist fractures within existing literature.

## Methods

We conducted a systematic review using the Preferred Reporting Items for Systematic Reviews and Meta-Analyses (PRISMA) checklist.^9^

## Collection of data

### Search strategy and criteria

The research question was formulated with keywords and concepts identified using the patient problem, intervention, comparison and outcome (PICO) process.^10^ In this review, the PICO framework was:

- Population: Study exclusively involving children (< 18 years) with a wrist fracture.
- Interventions/Comparison: Any intervention for management of acute wrist fractures.
- Outcomes: All outcomes.

Initial search-terms were then identified and exploded ensuring the inclusion of related terms and relevant synonyms. Multiple databases were used in order to ensure that a thorough search was carried out. These databases included the Cochrane Library, Ovid Medline, and SCOPUS. The search was then refined through the subsequent application of more specific terms and limitations. The suggested search terms for core outcome sets by Gargon et al^11^ were used as a guide. The eligibility criteria for this study is shown in Table I. For full details of the search see the supplementary text included within the appendix.

**Table I.** Inclusion and exclusion criteria for study.

Criteria type	Description
Inclusion criteria	Human participants
	Study exclusively involving children (< 18 years) with a wrist fracture
	Any involving interventions for the management of acute wrist fractures
Exclusion criteria	Studies that were not published in English
	Studies that were published greater than 15 years prior to the writing of this paper (prior to August 2003) to ensure that data are relevant to current practice
	Studies that included less than 20 cases to ensure papers selected for analysis were representative of the general population, and not published owing to novel findings
	Systematic reviews

The search was supplemented with thorough hand searching of the references of the articles retrieved for previously unfound published reports.

Four authors independently assessed all the titles and abstracts of published articles as a result of the initial search of the Cochrane Library, Scopus, and OVID Medline for eligibility. This search took place in August 2018. Full text reports were obtained where appropriate. Any disagreements upon the selection of studies was resolved via discussion. A fifth author was used to adjudicate where this was not possible.

### Data extraction

From each included publication one author extracted the following data: study type, demographic characteristics of the patients, including age, the number of patients, and the outcomes used within these studies, and the methods they used to measure these outcomes. This data was then checked independently by the second author.

### Data presentation

The data collected from the eligible studies was summarized in a textual format consistent with how the information was presented within the original articles.

Outcome terms were assigned to one of the core outcome domains from the revised Williamson-Clarke criteria of CoS outcomes (Table II).^12^

**Table II.** Overview of Williamson-Clarke criteria as modified by Dorman et al.^13^

Core area	Core domains	Examples
Adverse effects	Adverse events	Unintended consequences
Death	N/A	N/A
Physiological/ clinical	Musculoskeletal outcomes	Range of movement, grip strength etc.
Life impact	Physical/ social/ role/ emotional/ cognitive/ functioning Health-related quality of life (HRQoL) Delivery of care (does not refer to the resource delivery but instead includes satisfaction, patient preference, adherence, withdrawal, tolerability etc.)	Patient-reported outome measures, activities of daily living, pain intensity, patient experience and satisfaction, quality of life etc.
Resource use	Economic/ hospital/ need for intervention Societal burden	Length of stay, further surgery, physiotherapy etc.
Technical considerations	Technical/surgical considerations	Radiological measurements

A list of all potential outcomes was identified from the systematic review. Outcomes were listed both individually and by domain to aid interpretation. For each core domain area, the frequency of selection for each individual outcome measure was evaluated. The instruments used to capture each outcome and the time points at which they were utilized was also recorded.

### Quality assessment

The purpose of this study was to identify all outcomes reported irrespective of the study quality. While a poor-quality study may influence the outcomes of a conventional systematic review of clinical effectiveness, it will not have any influence on the types of outcomes being recorded. No formal quality assessment was therefore undertaken.

## Results

**Fig. 1:**
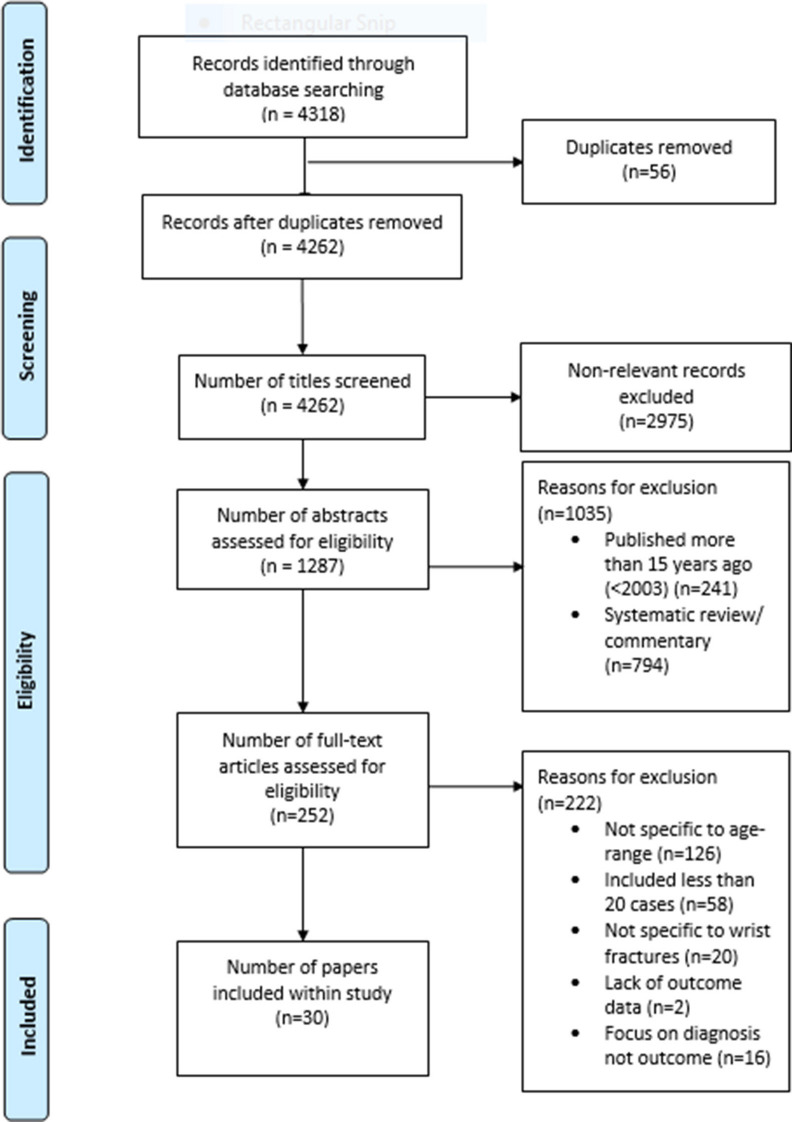
Flowchart of articles retrieved from searches of databases and reasons for exclusion.

The search identified 4,318 possible titles and abstracts; 56 duplicates were then removed. Initial review of the titles remaining articles excluded a further 2,975 articles, leaving 1,287 articles for assessment of eligibility. Review of the abstracts then excluded a further 1,035 papers, leaving 252 full papers for screening. After obtaining the full articles a further 222 studies were removed leaving 30 papers for inclusion within the study. Of note, no meta-analyses where deemed as eligible for this study. Searching references lists and conferring with experts added no further articles. This process is displayed in figure 1.

Information on the general characteristics and participants of the 30 selected studies is presented within Table III.

**Table III.** Presentation of results.

Paper	Age of participants at time of trauma (years)	Study design	Core Outcome Domains measured*
1^14^	Average: 8 Range: 3–14	Case series	**Technical/surgical considerations:** Radial inclination and palmar tilt of distal articular surface, ulnar variance and the residual angulation of the shaft of the radii. **Life impact:** Pain intensity. **Physiological/clinical:** Active range of movement of forearm and wrist (compared with contralateral side).
2^15^	Average: 9 Range: 1 to 16	Case series	**Technical/surgical considerations:** Radial inclination, ulna variance and palmar tilt as compared with the contralateral side. **Physiological/clinical:** Active range of movement of forearm and wrist (compared with contralateral side). **Life impact:** Pain intensity.
3^16^	Average: 8 5/12 Range: 4 to 12	Case series	**Technical/surgical considerations:** Magnitude of angulation of the radii and ulna. **Physiological/clinical:** Active range of movement of forearm (compared with contralateral side). **Life impact:** Patient satisfaction.
4^17^	Average: 9.9 Range: 3 to 15	Case series	**Technical/surgical considerations:** Residual angulation of the shaft of the radii and the distance of radial shortening. **Physiological/clinical:** Active range of movement of forearm (compared with contralateral side). **Adverse effects:** Rate of complication.
5^18^	Average: 8.5 Range: 9/12 to 15	Randomized controlled trial	**Life impact:** The willingness of the patient to use the immobilization again, daily pain scores, the duration of pain and the ability of the patient to return to employment/school.
6^19^	Average: 13.2 Range: 10 to 16	Cohort study	**Technical/ surgical considerations:** Fracture union time. **Physiological/clinical:** Active range of movement of forearm (compared with contralateral side). **Life impact:** Patient satisfaction.
7^20^	Average: 10.3 Range: 3.1–17.1 years	Case series	**Technical/surgical considerations:** Fracture union time and the residual angulation of the shaft of the radii. **Life impact:** Patient satisfaction. **Physiological/clinical:** Active range of movement of forearm (compared with contralateral side). **Adverse effects:** Rate of complication.
8^21^	Median:11 Range: 4 to 15	Case series	**Technical/surgical considerations:** Palmar tilt of the radii, the radial inclination, the residual angulation of the radial shaft and ulnar variance. **Life impact**: Pain intensity. **Physiological/clinical:** Active range of movement of forearm and wrist (compared with contralateral side) and the grip strength of the patient (% of contralateral side).
9^22^	Average: 9.3 Range: 2 to 6	Randomized controlled trial	**Life impact:** Level of difficulty involved in performing everyday tasks, patient satisfaction, the psychological status of the patient and pain Intensity. **Resource use:** Cost and the need for further healthcare.
10^23^	Average: 13.8 Range: 9.6 to 15.9	Case series	**Physiological/clinical:** Active range of movement of forearm and wrist (compared with contralateral side) and the grip strength of the patient (% of contralateral side). **Life impact:** Pain intensity and the ability of the patient to return to employment/school.
11^24^	Average: 13.7 Range: 10 to 16	Case-control study	**Technical/surgical considerations:** Fracture union time. **Life impact:** Patient satisfaction. **Physiological/clinical:** Active range of movement of forearm (compared with contralateral side). **Adverse effects:** Rate of complication.
12^25^	Average: 11 Range: 3.8 to 17.9	Case series	**Technical/surgical considerations:** Fracture union rate. **Life impact:** Patient satisfaction. **Physiological/clinical:** Active range of movement of forearm (compared with contralateral side). **Adverse effects:** Rate of complication.
13^26^	Average 11.6 Range: 5 to 17	Case series	**Technical/surgical considerations:** Radii and Ulna length. **Life impact:** Patient satisfaction. **Physiological/clinical:** Active range of movement of forearm (compared with contralateral side) and the circumference of forearm muscles compared to normal side. **Adverse effects:** Rate of complication.
14^27^	Average: 12.8 Range: 9.7 to 16.3	Case series	**Technical/surgical considerations:** Fracture union time, malunion and sclerotic changes of the scaphoid, as well as for malalignment or degenerative changes of the wrist. **Life impact:** The ability of the patient to return to employment/school, the level of difficulty involved in performing everyday tasks and pain intensity. **Physiological/clinical:** Active range of movement of forearm and wrist (compared with contralateral side) and the grip strength of the patient (% of contralateral side).
15^28^	Median: 14.5 Range: 8 to 18	Cohort study	**Life impact:** The ability of the patient to return to employment/school, the level of difficulty involved in performing everyday tasks and pain intensity. **Physiological/clinical:** Active range of movement of forearm and wrist (compared with contralateral side) and the grip strength of the patient (% of contralateral side).
16^29^	Average: 11 Range: 9 to 14	Case-control study	**Technical/surgical considerations:** Signs of radiocarpal joint degeneration, bone misalignment, cross union between the ulna and radii heterotrophic ossification, or any other residual bone deformity. **Physiological/clinical:** Active range of movement of forearm and wrist (compared with contralateral side), the grip strength of the patient (% of contralateral side) and cold sensitivity. **Life impact:** The ability of the patient to return to employment/school, the level of difficulty involved in performing everyday tasks, patient satisfaction and pain intensity. **Adverse effects:** Rate of complication
17^30^	Average: n/a Range: 6 to 15	Randomized controlled trial	**Life impact:** The ability of the patient to return to employment/school, the level of difficulty involved in performing everyday tasks and pain intensity.
18^31^	Average: 11 Range: 7 to 15	Case series	**Technical/ surgical consideration:** Bone healing time (fracture union time), alignment of radii and ulna (magnitude of angulation), and rotation of the radii. **Life impact:** The ability of the patient to return to employment/school, the level of difficulty involved in performing everyday tasks, patient satisfaction and pain intensity. **Physiological/ clinical:** Active range of movement of forearm and wrist (compared with contralateral side) and the grip strength of the patient (% of contralateral side).
19^32^	Average: 5.2 Range: n/a	Case series	**Technical/ surgical consideration:** Residual angulation of radii and ulna. **Resource use:** Time until management
20^33^	Average: 13.4	Case series	**Technical/ surgical consideration:** Fracture union time, magnitude of angulation, length of radii/ulna and distal radioulnar joint (DRUJ) subluxation. **Life impact:** Level of difficulty involved in performing everyday tasks. **Physiological/ clinical:** Active range of movement of forearm (compared with contralateral side).
21^34^	Average: 9.5 Range: 7 to 14	Case series	**Technical/ surgical consideration:** Translation and angulation of fracture. **Life impact:** Patient satisfaction. **Physiological/ clinical:** Active range of movement of forearm (compared with contralateral side) and the presence of any clinical deformity.
22^35^	Average: 9.6 Range: n/a	Case series	**Technical/ surgical consideration:** Magnitude of fracture angulation. **Life impact:** Level of difficulty involved in performing everyday tasks. **Physiological/ clinical:** Active range of movement of forearm (compared with contralateral side), the grip strength of the patient (% of contralateral side) and the presence of any clinical deformity.
23^36^	Average: 8.6 Range: n/a	Case series	**Technical/ surgical considerations:** Fracture union time, magnitude of angulation and rotation of fracture. **Adverse effects:** Complication rate.
24^37^	Average: 9.7 Range: 1.7–16.2	Case series	**Physiological/ clinical:** Active range of movement of forearm (compared with contralateral side). **Adverse effects:** Complication rate.
25^38^	Average:7.6 Range: 3 to 10	Case series	**Technical/ surgical considerations:** Ulna shortening, fracture union time and ulnar variance. **Physiological/ clinical:** Active range of movement of forearm (compared with contralateral side). **Adverse effects:** Rate of complication.
26^39^	Average: 14.1 Range: 12.1 to 17.6	Case series	**Technical/ surgical considerations:** Fracture union time and residual angulation. **Physiological/ clinical:** Active range of movement of forearm (compared with contralateral side).
27^40^	Average: 11.5 Range: 7 to 14	Case series	**Technical/ surgical considerations:** Fracture union time and radial angulation. **Physiological/ clinical:** Active range of movement of forearm (compared with contralateral side). **Adverse effects:** Rate of complication
28^41^	Average: 8 Range: 1 to 15	Case series	**Technical/ surgical considerations:** Translation and angulation of fracture. **Adverse effects:** Rate of complication.
29^42^	Average: n/a Range: 5 to 12	Randomized controlled trial	**Technical/ surgical considerations:** Magnitude of angulation of fracture **Life impact:** Pain intensity and patient satisfaction. **Physiological/ clinical:** Active range of movement of forearm and wrist (compared with contralateral side) and the grip strength of the patient (% of contralateral side).
30^43^	Average: 8.7 Range: n/a	Case series	**Technical/ surgical considerations:** Magnitude of angulation of the fracture **Physiological/clinical:** Active range of movement of forearm (compared with contralateral side), the grip strength of the patient (% of contralateral side) and the presence of any clinical deformity. **Resource use:** Further medical/ surgical needs

* A full description of the method of outcome assessment is available online as an appendix (Online Table I).

**Fig. 2:**
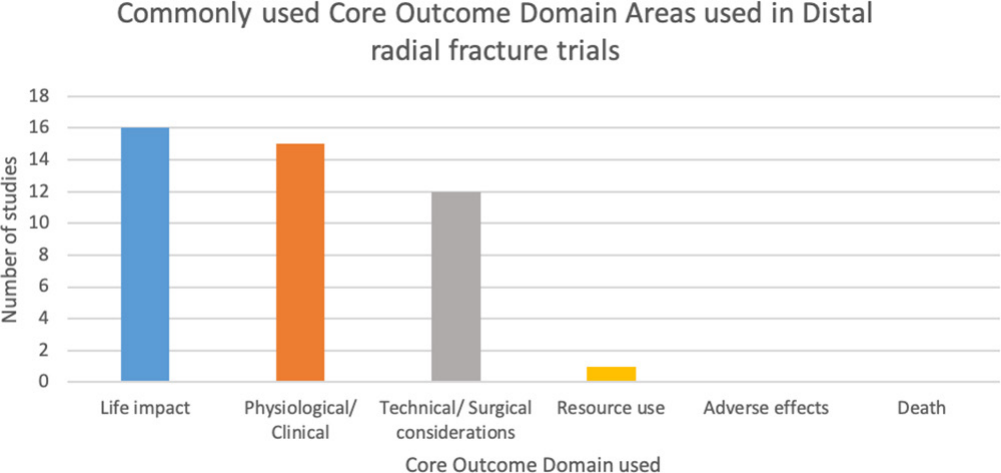
Summary of core outcome domains used within the studies included in this review.

The most common outcome domain measured was physiological/ clinical, with 24/30 studies using at least one measurement pertaining to this (Figure 2). The next most commonly used domains were Technical/Surgical, Life Impact and Adverse Effects, with 23, 21 and 11 of 30 studies respectively, using such measures. All studies used measures from one of these 4 domains with 3 studies recording any outcomes pertaining to the domain “Resource Use”.

**Fig. 3:**
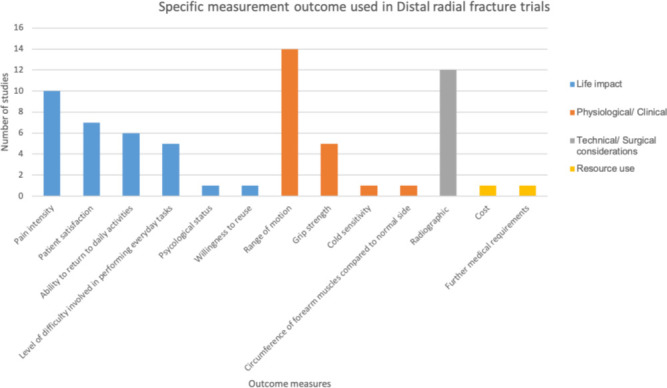
Summary of the measurements used to assess outcome within the studies included in this review.

**Fig. 4:**
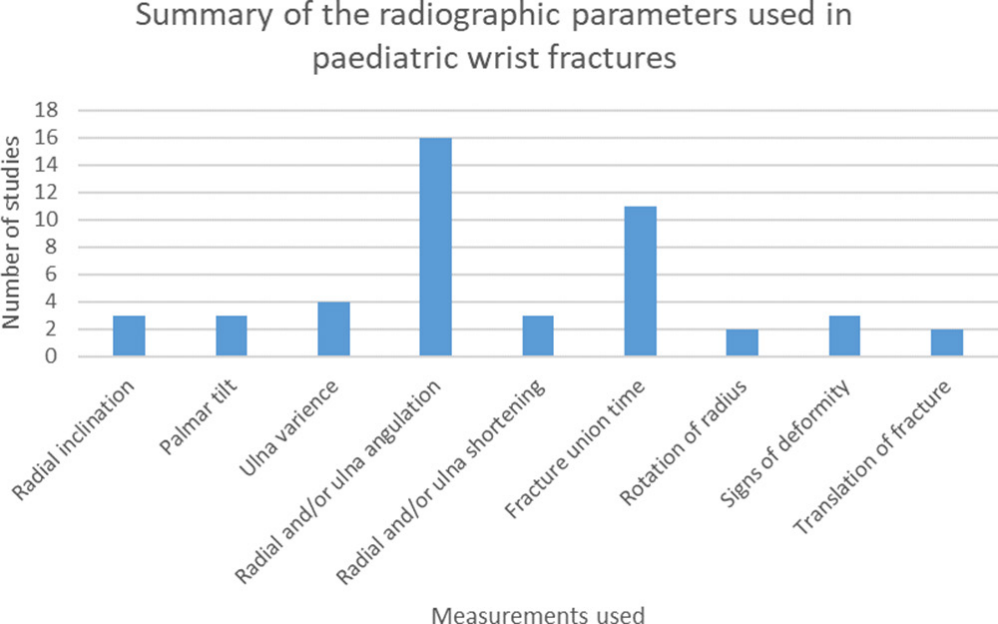
Summary of radiological parameters used within the studies included in this review.

In total there were 16 different measures used in the selected studies, with many different tools to assess these (Figure 3). The most common measures used were range of movement, a broad array of “radiological parameters (Figure 4)” and pain intensity. Cost-effectiveness, the need for further interventions, psychological assessment, and likelihood to reuse the service, cold sensitivity, duration of treatment and circumference relative to the patient’s other forearm were all used in one study each.

## Discussion

It is clear that there is significant heterogeneity in outcomes reported in the management of paediatric wrist fractures. Many different outcome domains were used, with a variety of different outcome measures utalised within each domain. Further to this, even where the same outcomes were used, commonly different measurement instruments within the assessment of outcome. The vast majority of studies included measures pertaining to the outcome domains of ‘Life Impact’ (i.e. pain or the ability to function normally), ‘Physiological/ Clinical’ (i.e. the range of wrist movement) and Technical/Surgical Considerations (i.e. a surgeon’s assessment of how close the wrist is restored to normal anatomy). However, within each domain, there was almost no agreement on any of the measurements to be used, such that there were 5 different ways of measuring pain, 7 different ways of measuring function and 9 different radiological parameters used. Such variation limits the ability to compare outcomes for different interventions between studies, or the performance of different health care providers.

Measures of Life Impact domain were most common, with instruments assessing a child’s perception of pain/ recovery, satisfaction and return to daily function. These were largely assessed through patient-reported outcomes (PROs), which are generally considered the outcomes most important to patients. Pain may be considered the simplest thing to record, as techniques for pain measurement have been extensively researched in children, and a number of high-quality systematic reviews exist to outline the optimal means of measurement in children.^44,45^ However, pain was recorded in 5 different ways; several of which were not reliable or valid measures in this population. Functional PROs are more complex, seeking to quantify functional capacity, such as the ability to return to perform activities of daily living, and are more subjective.^46,47^ PROs used included the Modified Mayo Wrist Score (MMWS), Disabilities of the Arm, Shoulder, and Hand (DASH) Score and Activities Scale for Kids (ASKp). However, there is significant concern about the validity of these measures in the child population. ASKp has been validated within children aged 5 to 15,^48^ but there no such validation for the use of DASH and MMWS, which were developed for use in adults. Furthermore, these PROs do not appear to have ‘face-validity’ in a child population with questions pertaining to sexual function, ‘heavy household chores’ and employment status. While ASKp is valid in this population, it was only used in 2 papers, though the sensitivity for change among children with wrist injuries in unknown.

Surgical/technical outcomes were the second most commonly used domain, typically through measurements of radiological parameters. However, a large number of measurement tools were used, with little consistency between studies; including radial inclination, palmar tilt of articular surface, ulnar variance, and residual angulation of the shaft of radii / ulna, length of the radii and fracture union time. There was no standardization of the timing of radiological measures.

Furthermore, although radiological parameters are widely used as outcomes, there is little to no evidence that such outcomes correlate with important functional outcomes. However in adults it has been shown that these parameters correlate poorly with patient reported outcomes.^49,50^ While this is not in a paediatric population it still raises significant concerns with the use of radiological outcomes.

The domain “Adverse Effects” of therapeutic interventions were described in 11/30 papers. There was no standardized way of reporting, and incorporating complications into overall outcome. The domain “Resource Use” was reported in only 3 studies with instruments being used to measure the cost of the primary therapeutic intervention as well as the need for further medical interventions.

Due to the wide variability within the outcomes selected throughout these studies it is clear that a consensus needs to be reached among health care professionals regarding the most important domains, outcome measures and outcome measurement instruments. This is perhaps most important for PROs, where there is a significant desire to record these outcomes by surgeons, though where invalid measurement instruments are frequently used. The type of PROs used may however be on the cusp of changing, as the US National Institutes of Health (NIH) have an established Patient-Reported Outcomes Measurement Information System (PROMIS) Roadmap initiative. There is a specific PROMIS Upper Limb measurement instrument for use in children, which has both patient-reported and proxy-reported versions, and is valid from five years old. PROMIS tools for children have been extensively developed and tested within a population of 4,636 children by experts in the development of PROs, with extensive validation.^51,52^ The existence of a valid upper limb score for children is likely to standardize the way that upper limb function is recorded, though similar standardization will be necessary for other elements of life-impact, such as pain.

There are inherent limitations with reviewing outcomes within existing literature, with studies selected particularly prone to reporting bias where authors report only outcomes with favourable results. In order to minimize the effect of this bias, this review focused only upon the outcomes used rather than the outcome result.

We collected information from a large number of studies, excluding small studies which may introduce outcome measures most relevant in a few novel cases. The quality of the studies reported, or quality of the results obtained, are therefore less relevant to this review which is most concerned with which outcomes were felt to be important, and measured accordingly.

This review documents the range of outcome domains, measures and tools within studies of paediatric wrist factures. The inconsistencies in outcomes measured between studies makes meta-analyses difficult to interpret. Consensus regarding the most important outcomes, the COS, is urgently required. In doing this, it is important to elicit the opinions of children, parents, and carers, as well as clinicians, to ensure important outcomes are universally recorded. Until there is a consistent approach to the study of this common injury, there will be ongoing heterogeneity between studies, with research unable to elicit answers to the key questions.
